# Hypoxia Is Not a Main Stress When *Mycobacterium tuberculosis* Is in a Dormancy-Like Long-Chain Fatty Acid Environment

**DOI:** 10.3389/fcimb.2018.00449

**Published:** 2019-01-09

**Authors:** Patricia Del Portillo, Lázaro García-Morales, María Carmen Menéndez, Juan Manuel Anzola, Juan Germán Rodríguez, Addy Cecilia Helguera-Repetto, Miguel A. Ares, Rafael Prados-Rosales, Jorge A. Gonzalez-y-Merchand, María Jesús García

**Affiliations:** ^1^Departamento de Biotecnología Molecular y Biología Computacional y Bioinformática, Corporación CorpoGen, Bogotá, Colombia; ^2^Departamento de Biomedicina Molecular, Centro de Investigación y de Estudios Avanzados del Instituto Politécnico Nacional (CINVESTAV), Ciudad de México, Mexico; ^3^Departamento de Medicina Preventiva, Facultad de Medicina, Universidad Autónoma de Madrid, Madrid, Spain; ^4^Departamento de Inmunobioquímica, Torre de Investigación, Instituto Nacional de Perinatología Isidro Espinosa de los Reyes, Ciudad de México, Mexico; ^5^Unidad de Investigación Médica en Enfermedades Infecciosas y Parasitarias, Centro Médico Nacional Siglo XXI, Instituto Mexicano del Seguro Social, Ciudad de México, Mexico; ^6^Center for Cooperative Research bioGUNE (CICbioGUNE), Bizkaia Technology Park, Derio, Spain; ^7^Departamento de Microbiología, Escuela Nacional de Ciencias Biológicas, Instituto Politécnico Nacional, Ciudad de México, Mexico

**Keywords:** *Mycobacterium tuberculosis*, lipid environment, dormancy, hypoxia, gene expression, RNA-sequencing, stress response

## Abstract

The capacity of *Mycobacterium tuberculosis* (*Mtb*) to sense, respond and adapt to a variable and hostile environment within the host makes it one of the most successful human pathogens. During different stages of infection, *Mtb* is surrounded by a plethora of lipid molecules and current evidence points out the relevance of fatty acids during the infectious process. In this study, we have compared the transcriptional response of *Mtb* to hypoxia in cultures supplemented with a mix of even long-chain fatty acids or dextrose as main carbon sources. Using RNA sequencing, we have identified differential expressed genes in early and late hypoxia, defined according to the *in vitro* Wayne and Hayes model, and compared the results with the exponential phase of growth in both carbon sources. We show that the number of genes over-expressed in the lipid medium was quite low in both, early and late hypoxia, relative to conditions including dextrose, with the exception of transcripts of stable and non-coding RNAs, which were more expressed in the fatty acid medium. We found that *sigB* and *sigE* were over-expressed in the early phase of hypoxia, confirming their pivotal role in early adaptation to low oxygen concentration independently of the carbon source. A drastic contrast was found with the transcriptional regulatory factors at early hypoxia. Only 2 transcriptional factors were over-expressed in early hypoxia in the lipid medium compared to 37 that were over-expressed in the dextrose medium. Instead of Rv0081, known to be the central regulator of hypoxia in dextrose, Rv2745c (ClgR), seems to play a main role in hypoxia in the fatty acid medium. The low level of genes associated to the stress-response during their adaptation to hypoxia in fatty acids, suggests that this lipid environment makes hypoxia a less stressful condition for the tubercle bacilli. Taken all together, these results indicate that the presence of lipid molecules shapes the metabolic response of *Mtb* to an adaptive state for different stresses within the host, including hypoxia. This fact could explain the success of *Mtb* to establish long-term survival during latent infection.

## Introduction

It is estimated that more than two billion people in the world have latent tuberculosis infection (LTBI), an asymptomatic and non-infectious form of the disease where the causative agent, *Mycobacterium tuberculosis* (*Mtb*), is primarily in a dormant state (Dye et al., 1999; Getahun et al., 2015). Several studies have shown that the dormant bacillus must face different hostile microenvironments within the host to survive, including hypoxia, lack of nutrients, and acidic pH (Deb et al., 2009; Flentie et al., 2016). These wide spectra of host-induced stresses could explain the existence of several subpopulations of the tubercle bacilli, with diverse physiological states and consequently, with heterogeneous metabolic activities (Prosser et al., 2017). Importantly, the dormant *Mtb* retains their capacity for reactivation and developing active tuberculosis (TB) (Veatch and Kaushal, 2018).

Different *in vitro* models have been developed to study dormancy in *Mtb* and although, it is clear that an *in vitro* model cannot reproduce the complex interaction between *Mtb* and the human immune system, such models are useful to decipher the metabolic changes that mycobacteria undergo to remain alive for long periods of time in its human host. Most studies adopt the *in vitro* Wayne and Hayes method to study dormancy in *Mtb*, which mimic the hypoxic conditions inside a granuloma (Wayne and Hayes, 1996). In this model, oxygen is gradually eliminated from an exponential *Mtb* culture, originally in Dubos medium with dextrose as the carbon source. These conditions allow *Mtb* to adapt to what those authors called non-replicating persistence (NRP) states 1 and 2, with a remaining of 1 and 0.06% of oxygen, respectively.

Alternatively, the *Mtb*'s lipid metabolism has emerged as an important factor to consider in the study of LTBI (Mdluli et al., 2015; Tobin, 2015; Warner et al., 2015). For instance, the metabolism of triacylglycerols (TAG) is necessary for the survival of *Mtb* inside foamy macrophages during LTBI (Santucci et al., 2016). Recently, several studies have focused in the relevance of cholesterol during *Mtb* infection (Chang et al., 2009; García et al., 2012; Mclean et al., 2012; Soto-Ramirez et al., 2017). In addition, we have shown that besides sterols, long-chain fatty acids (LC-FA) are also key participants as components of the *Mtb* metabolism inside the host since *Mtb* cultured at stationary phase in the presence of LC-FA induced a dormant phenotype (i.e., accumulation of lipid droplets, increase of drug tolerance and low metabolic activity) (Rodríguez et al., 2014). In agreement with our previous results, Nandy and co-workers showed that the metabolism of oleic acid leads to a reductive cytosol in *Mtb*, which counteracts oxidative stress in a caseous-necrotic environment displayed by adipocytes (Nandy et al., 2018). These results strongly suggest that *Mtb* is adapted to a lipid environment *in vivo*.

The bacterial sensing of different environmental stimuli leading to physiological and phenotypical changes generate a response that switches the transcriptional apparatus throughout the expression of a complex net of regulatory gene systems. These processes also occur in *Mtb*, a bacterium with a remarkable number of regulatory systems in relation to its genome size (Cole et al., 1998). *Mtb* contains a large repertoire of transcriptional regulators, 13 of which are the well-known sigma factors, SigA to SigM (Manganelli et al., 2004). Sigma factors respond to different environmental stimulus, for example, *sigE*, a factor essential for *Mtb* virulence, is over-expressed together with *sigH*, during persistent infection and reactivation (Veatch and Kaushal, 2018). Over-expression of *sigB, sigE*, and *sigH* has also been documented in the hypoxic environment either in the presence of dextrose or lipids (Rodríguez et al., 2014; Aguilar-Ayala et al., 2017; Pisu et al., 2017). Besides sigma factors, other additional 50 transcriptional factors (TFs) have been related to hypoxia (Galagan et al., 2013). Among them, the three-component system DosRS/T is one of the most studied (Dutta and Karakousis, 2014). Other relevant regulators involved in the adaptation to hypoxia are members of the enduring hypoxic response (EHR) (Rustad et al., 2008) and the *Rv0081* gene, the central regulator of hypoxia described by Galagan and co-workers (Galagan et al., 2013). Notably, the contribution of Rv0081, EHR, and DosRS/T during the growth of *Mtb* in the presence of lipids is mostly unknown. In our previous transcriptional study, we found that *Rv0081* together with some *dosR*-regulated genes are over-expressed during a dormancy-related stationary phase in the presence of LC-FA (Rodríguez et al., 2014). Moreover, an increase in the expression of the *dosR* regulon was reported to occur during the exponential phase of *Mtb* growth in the presence of cholesterol, while its expression decreased during the NRP1 (Aguilar-Ayala et al., 2017).

In addition to TF, mycobacterial small RNAs (sRNAs) have also been shown to modulate the response to environmental changes (Arnvig and Young, 2009). Mycobacteria have more than 200 sRNAs, most of which are non-coding RNAs (ncRNAs) (Haning et al., 2014). The *Mtb* most highly expressed ncRNA is MTS2823 (ncRv13661A according to the new nomenclature Lamichhane et al., 2013) and was found to be upregulated under different conditions, including growth of *Mtb* in the presence of LC-FA as a carbon source (Rodríguez et al., 2014). In addition, it is the most expressed ncRNA in both exponential and stationary phases of growth and in the lungs of infected mice (Arnvig and Young, 2012); on the other hand, the ncRNA MTS1338 (ncRv1734A according to the new nomenclature Lamichhane et al., 2013) has been found to be associated to the over-expression of the *Mtb dosR* regulon (Moores et al., 2017).

*Mtb* can also respond to stress microenvironments throughout the differential expression of toxin-antitoxin (TA) biological systems (Gerdes and Maisonneuve, 2012). Under several stresses the antitoxin could be degraded and the toxin could block essential cellular processes, leading to a low metabolic activity of the bacteria (Fernandez-Garcia et al., 2016). To date, a total of 80 TA systems have been identified in the *Mtb* genome (Sala et al., 2014; Slayden et al., 2018). Of note, some of them were found to be upregulated during hypoxia (Ramage et al., 2009) and up to 11 of them were upregulated during starvation (Sala et al., 2014). Most of these systems were identified as Type II-TA systems, belonging to the VapBC family (stands for virulence associated proteins), with the antitoxin blocking its corresponding toxin by a direct fusion of protein-protein (Fernandez-Garcia et al., 2016). Recently, it was shown that the exposure of *Mtb* to cholesterol induces over-expression of some *vapBC* genes, in particular, the complete modules *vapBC22* and *vapBC9*, regardless of the environmental level of oxygen (Aguilar-Ayala et al., 2017).

The aim of this work was to gain a deeper understanding of the pathway that *Mtb* uses to adapt to hypoxia by analyzing both NRP1 and NRP2 phases of dormancy in the presence of LC-FA. Our results revealed that the presence of LC-FA induces a dormancy-like state in *Mtb* that seems to buffer the hypoxic response as shown by the minor regulatory change observed during the entrance to hypoxia in that lipid environment.

## Materials and Methods

### Bacterial Strain and Hypoxic Culture Conditions

*Mycobacterium tuberculosis* H37Rv was grown in Dubos medium (Difco Dubos Broth Base, USA) and supplemented with either: (1) 0.2% dextrose (D), or (2) long chain fatty acids (F) (oleic acid, stearic acid and palmitic acid at a final concentration of 0.001% each) as main carbon sources at 37°C. Exponential phase cultures at an OD_600_ = 0.4 were obtained at day 7 (with dextrose) and day 8 (with FA), according to the growth curve published previously by our group (Rodríguez et al., 2014). Exponential cultures were submitted to hypoxic conditions by leaving a ratio of air volume to liquid medium of 0.5 in flasks with tightly sealed caps. Cultures were agitated with a 50 mm teflon-coated magnetic stirrer bar at 70 rpm, as previously reported (Wayne and Hayes, 1996). NRP1 and NRP2 states were defined, according to Wayne and Hayes (1996), with a parallel culture supplemented with methylene blue (1.5 μg/mL), which indicates approximate oxygen depletion. In this way, the NRP1 phase (fading of methylene blue) was reached at day four in both, fatty acids and dextrose medium (FNRP1 and DNRP1); and NRP2 (complete decolorization of methylene blue) was reached at day eight (FNRP2 and DNRP2). RNA isolation and CFU/ml quantification were carried out 24 h after reaching the corresponding NRP phase.

### RNA-Seq

RNA from each growing condition (exponential, NRP1 and NRP2) was isolated as previously described (Rodríguez et al., 2014). Briefly, cells were harvested and resuspended in guanidium chloride buffer (6 M guanidinium chloride, 0.1% Tween 80, 1 mM 2-mercaptoethanol, 10 mM EDTA) and lysed mechanically in a FastPrep (Thermo Scientific). Nucleic acids were purified using phenol- chloroform- isoamyl alcohol and RNA was selectively precipitated with absolute ethanol and washed three times with Trizol reagent (Invitrogen). RNA integrity was estimated with bioanalyzer (Agilent Technologies) and quantified by Nano-drop ND 1000 (Thermo Scientific). RNASeq libraries were prepared as previously describe (Rodríguez et al., 2014). Briefly, total RNA was fragmented, submitted to poly A tailing and end repairing (NEB reagents, USA). Strand-specific labeling was carried out by ligation of a 5′ hybrid DNA-RNA primer. Reactions for the synthesis of the first cDNA strand was carried out with Super Script II reverse transcriptase (Invitrogen, USA), Illumina's poly T primer and dNTPs (20 mM). Illumina adaptors and barcodes were ligated by PCR following manufacturer instructions and pair end sequencing was performed on Illumina HiSeq sequencer.

### RNA-Seq Data and Statistics Analysis

After trimming, those sequences with quality scores over 30 (Illumina 1.3+) and a minimal length of 50 bases were used for the analysis. Quality assessment of the reads was done using the FASTX toolkit v. 0.0.13 (http://hannonlab.cshl.edu/fastx_toolkit/index.html). The reads were mapped against the *Mtb* H37Rv reference genome (accession number NC 000962.2) using Bowtie v. 0.12.7 (Langmead et al., 2009). Resulting SAM files were used with the Tophat package (Langmead et al., 2009) to compute gene expression, differential gene expression and statistical significance. Gene expression was computed as RPKM (Reads Per Kilobase Million of mapped Reads). Statistical significance was determined on a binary basis between conditions by False Discovery Rate (FDR). We defined genes with a FDR of <0.05 as over-expressed.

### qRT-PCR of Selected Genes

The total transcripts of selected genes were measured by real-time qRT-PCR in a LightCycler 480 Instrument (Roche). Quantification was performed with gene-specific primers (see Supplementary Table 1) and SYBR green (Molecular Probes, Inc.). Samples were subjected to 40 cycles of amplification (denaturation at 95°C for 30 s, specific annealing temperature for 20 s, and extension at 72°C for 30 s) with a final extension at 72°C for 5 min. To ensure that the fluorescence levels detected were due to the amplification of a specific product, a melting curve analysis was performed. Absolute quantification was carried out by obtaining a standard curve for each set of primers according to 10-fold dilutions of by known amounts of *Mtb* H37Rv chromosomal DNA (1,000, 10,000, 100,000, and 1,000,000 theoretical copies). Crossing point values were interpolated to standard curve to obtain gene expression (number of gene copies per μg of RNA). Normalization of these data was performed by using 16S rRNA expression levels.

### Nucleotide Sequence Accession Number

The RNAseq data sets have been deposited in NCBI's Gene Expression Omnibus (Edgar et al., 2002) under accession number GSE119225.

## Results

### The Global Transcriptome of *M. tuberculosis* in the Fatty Acid Model of Hypoxia

To gain insight into the adaptation of *Mtb* to hypoxia in a lipid environment we performed a transcriptomic analysis of *Mtb* following the Wayne and Hayes model (Wayne and Hayes, 1996) with growing conditions that included dextrose or LC-FA as main carbon sources (Rodríguez et al., 2014). NRP1 phase was reached at day four after exponential phase in both fatty acids and dextrose medium (FNRP1 and DNRP1); NRP2 phase (complete fading of methylene blue) was reached at day eight (FNRP2 and DNRP2) (see Materials and Methods). Table 1 shows the global results obtained for each condition. The sequences retained after cleaning were between 10.34 and 18.31 million reads, which cover the *Mtb* genome as indicated by the plateau reached in the saturation curves when mapping the reads against the *Mtb* genome (data not shown). To facilitate the analysis, and allow the comparison with previous studies, the data were normalized as reads per kilobase per million reads (RPKMs). As expected, we observed a reduction in about 20 to 35% in reads mapping to coding sequences (CDS) in both, the dextrose hypoxia model (DNRP1 and DNRP2) and the fatty acid hypoxia model (FNRP1 and FNRP2) (Table 1). This reduction was accompanied with an increase of reads of intergenic regions (IGRs). The high mapping reads against IGRs in the presence of fatty acids, especially in FNRP2, is similar to the IGRs expression observed in the stationary phase of bacilli growing in LC-FA (Rodríguez et al., 2014) when bacteria develop the dormant phenotype.

**Table 1:** *M. tuberculosis* hypoxic transcriptomes in the presence of dextrose and LC-FA.

	**No (%) of reads in following conditions**					
**Reads**	**DE**	**DNRP1**	**DNRP2**	**FE**	**FNRP1**	**FNRP2**
All	13.12	14.46	13.88	18.31	15.74	10.34
All mapped	8.17	8.11	9.35	9.56	11.19	4.78
**MAPPED WITHOUT rRNA**						
Mapped to CDS	0.52 (71.3)	0.38 (48)	0.62 (36)	0.28 (61.6)	0.35 (36)	0.15 (15)
Mapped to IGRs	0.17 (22.4)	0.40 (50)	0.87 (50.5)	0.15 (32.9)	0.6 (60)	0.7 (82)
Mapped to antisense CDS	0.05 (6.4)	0.05 (5.0)	0.02 (6.7)	0.02 (5.4)	0.04 (4.4)	0.001 (1)

*Total numbers (in millions) of reads determined under the different conditions (upper part); and numbers (percentages) of reads determined by excluding those corresponding to rRNA (lower part) are shown. Data from DE and FE conditions are taken from a previous study (Rodríguez et al., 2014). DE, Dextrose exponential; DNRP1, Dextrose early hypoxia; DNRP2, Dextrose late hypoxia; FE, LC-FA exponential; FNRP1, LC-FA early hypoxia; FNRP2, LC-FA late hypoxia*.

**Figure 1:**
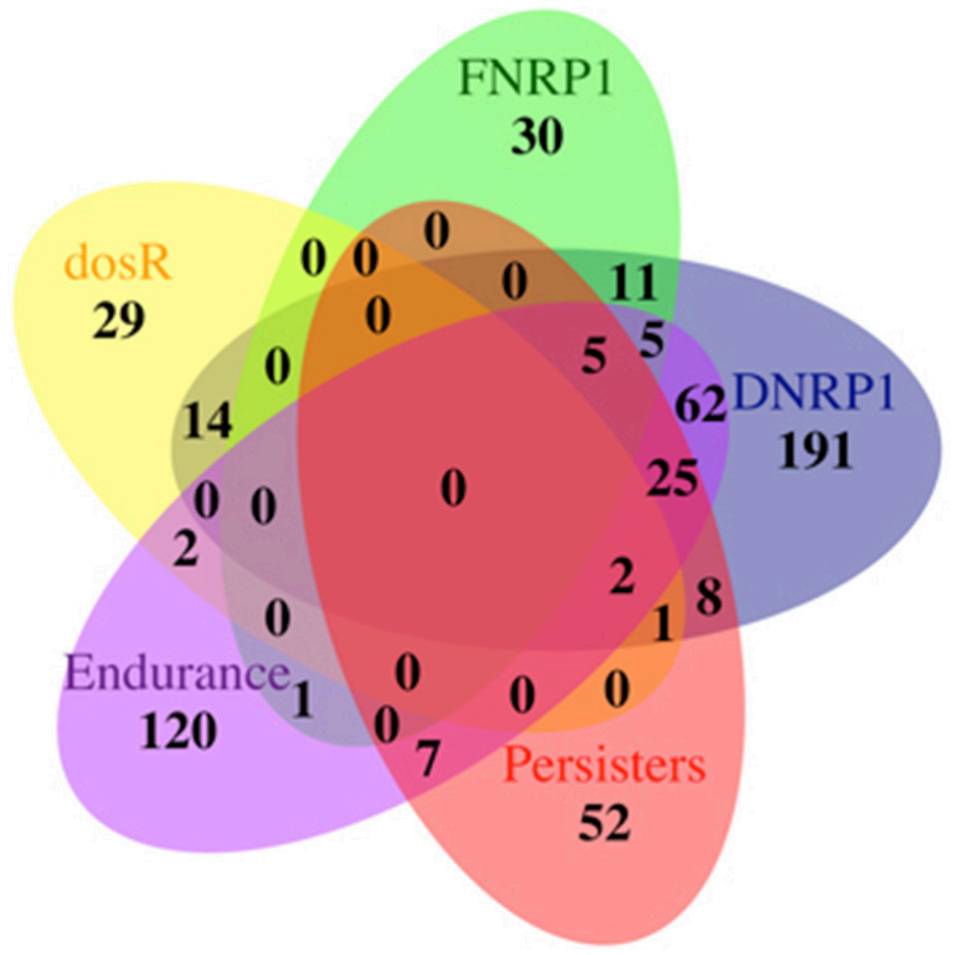
Venn diagram showing the number of statistically significant expressed genes by *Mtb* grown in conditions including early hypoxia in the presence of dextrose (DNRP1) and long-chain fatty acids (FNRP1); Endurance (EHR) regulon (Rustad et al., 2008), *dosR* regulon (Voskuil et al., 2004) and persister cells in antibiotics (Keren et al., 2011).

We next searched for parallels between our data and that of other hypoxia models, including the DosR and EHR (Voskuil et al., 2004; Rustad et al., 2008) as well as a persistence condition developed in the presence of antibiotics (Keren et al., 2011) (Figure 1). For these comparisons, we used data from early hypoxic stages (DNRP1 and FNRP1). Genes found to be shared between conditions are indicated in the Supplementary Table 2. As expected, data from DNRP1 had a higher number of genes in common with those from the *dosR* regulon (Voskuil et al., 2004) and EHR (Rustad et al., 2008), with 17 genes and 98 genes in common, respectively; Only two genes were found to be common to all these three conditions. Up to 32 genes were commonly over-expressed among DNRP1, persistence and EHR; and near 40 genes were common among persisters and either EHR or DNPR1 (Figure 1 and Supplementary Table 2).

Interestingly, we found that 5 out of 10 genes that were common among DNRP1/EHR/FNRP1 were also over-expressed in the persister cells (Supplementary Table 1). Those five genes are *Rv0251c, Rv1221, Rv2050, Rv2694c*, and *Rv2745c* encoding for the heat shock protein hsp, the alternative sigma factor SigE, the RNA polymerase-binding protein RbpA, the conserved protein Rv2694c and the transcriptional regulatory protein ClgR, respectively. These results suggest common pathways of adaptation to stresses conditions represented by hypoxia and persistence in antibiotics. Surprisingly, we could not find genes in common between FNPR1 and *dosR* regulon, while 11 and 21 genes were shared respectively between FNRP1 and the EHR and DNRP1 datasets. Of those, the gene *Rv2137c*, encoding a conserved hypothetical protein, was unique between FNRP1 and EHR dataset.

Taking into consideration that in our previous study 27 genes belonging to the *dosR* regulon were found to be over-expressed in the stationary phase cultures in the presence of LC-FAs (Rodríguez et al., 2014) these results suggest that the *dosR* regulon response was further unchanged when *Mtb* enter into hypoxia under our model of LC-FA lipid environment. Aguilar-Ayala and co-workers found similar results using the same LC-FA condition plus cholesterol as carbon source (Aguilar-Ayala et al., 2017) reinforcing our findings. Notably, no common genes were found between the five conditions compared (Figure 1 and Supplementary Table 2).

### Functional Categories Participating in the Fatty Acid Model of Hypoxia

**Figure 2:**
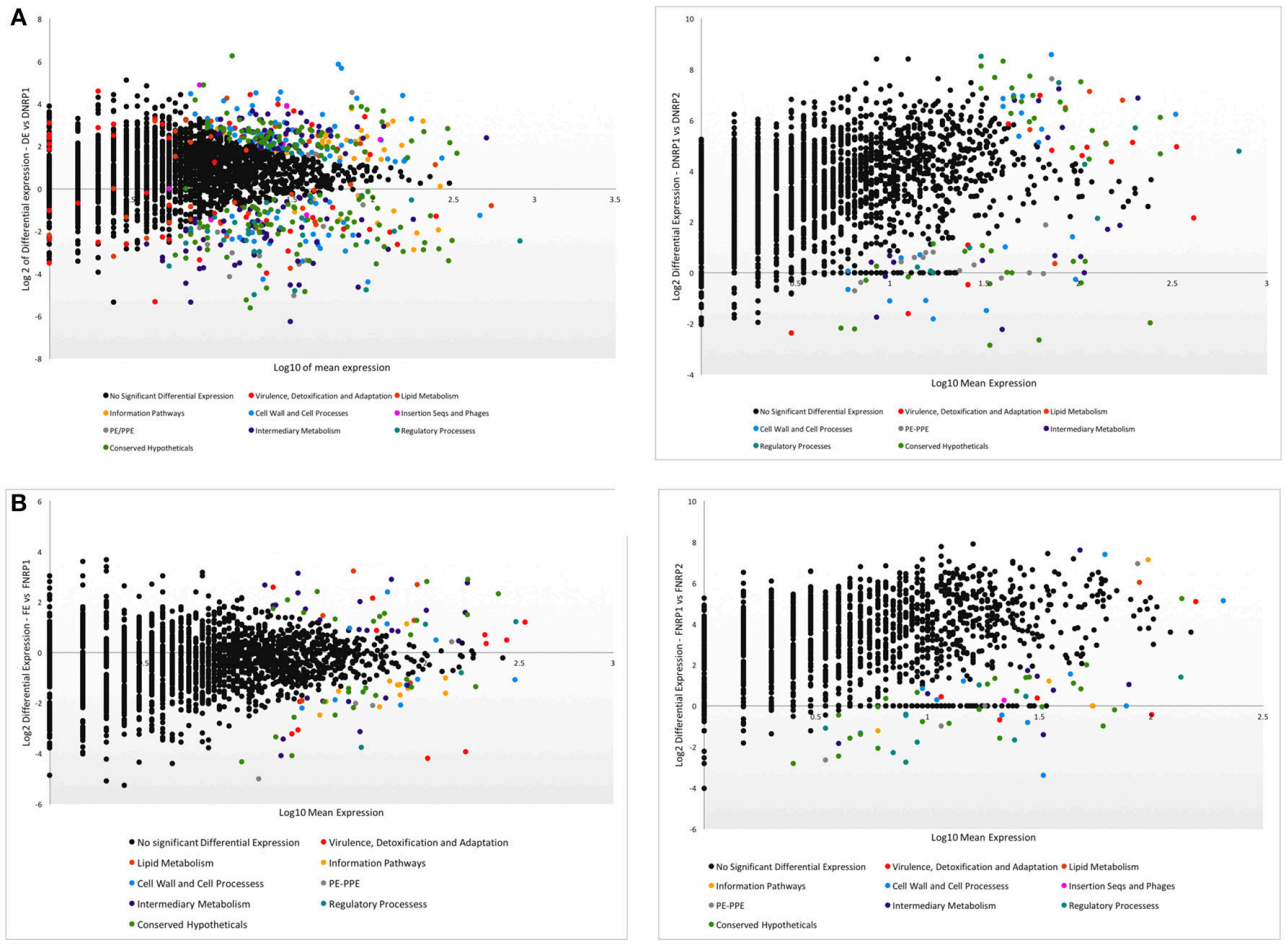
Volcano plots showing the functional categories of genes differentially expressed during the adaptation to hypoxia under different carbon sources. **(A)** Hypoxia in dextrose as carbon source. Left panel, DE compared to DNRP1; right panel, DNRP1 compared to DNRP2. **(B)** Hypoxia in LC-FA as carbon source. Left panel, FE compared to FNRP1; right panel, FNRP1 compared to FNRP2.

To determine changes in the metabolic function of *Mtb* during its adaptation to hypoxia, we compared functional categories of differentially expressed genes by *Mtb* grown in the three conditions including dextrose (namely, DE, DNRP1, and DNRP2) as well as the three conditions including LC-FA (FE, FNRP1, and FNRP2), as corresponding carbon sources (Figure 2 and Supplementary Table 3).

- **Dextrose cultures**. We found that when *Mtb* enters in early hypoxia in the presence of dextrose (DNRP1), expression of genes belonging to both information pathways and cell wall and cell processes functional categories are reduced relative to the exponential and normoxic phase (DE). These results suggest a decreasing level of the basic cell activity during adaptation to low oxygen. Conversely, an increase in the expression of genes of regulatory proteins and *PE/PPE* genes of 20 and 10%, respectively, was observed (Figure 2A). The higher number of regulatory proteins suggests an adaptation process of *Mtb* to tight their metabolic activity under hypoxia in a dextrose environment. Upon extended hypoxic stress (DNRP2), we observed a global reduction in gene expression of all functional categories compared to early hypoxia (DNRP1), indicating a sharp and global decrease of the metabolic activity of the bacilli under such circumstances (Figure 2A).
- **LC-FA cultures**. The overall number of the differential expressed genes was lower in the LC-FA media, relative to similar conditions with dextrose. During the entrance to hypoxia in a LC-FA environment (FNRP1) genes belonging to functional categories of intermediary metabolism and virulence, detoxification, and adaptation showed a two-fold decrease in expression. The decreased number of genes in the latter category could indicate that the bacilli are, at some extent, protected from the stress represented by hypoxia when LC-FA is present. Over-expressed functional categories in FNRP1 *vs*. FE were information pathways and PE/PPE genes (Figure 2B). Similar to the growing conditions including only dextrose, we found an increase in the expression of genes belonging to regulatory proteins during the adaptation from early to late hypoxia in the fatty-acid environment (FNRP1 *vs*. FNRP2) (Figure 2B). Of note, four out of six genes from this functional category over-expressed in FNRP2 (compared to FNRP1) were also over-expressed in DNRP1 relative to DE (Supplementary Table 3). This result suggests similarities at the regulatory level between the adaptation to late hypoxia in LC-FA and the adaptation to early hypoxia in dextrose (FNRP2 and DNRP1 respectively).

According to our results, about 10% of the *PE/PPE* genes showed over-expression during the entrance to early hypoxia independently of the carbon source (Figures 2A,B). Sixteen *PE/PPE* genes were over-expressed in DNRP1 *vs*. DE, meanwhile only three were over-expressed in FNRP1 *vs*. FE and *PPE31* (*Rv1807*) common to both conditions. *PPE31* has been identified as an essential gene for *Mtb in vivo* (Fishbein et al., 2015) and, according to our data, could also play some role in the adaptation to early hypoxia of the tubercle bacilli.

### Transcription Factors Involved in the Fatty Acid Model of Hypoxia

**Figure 3:**
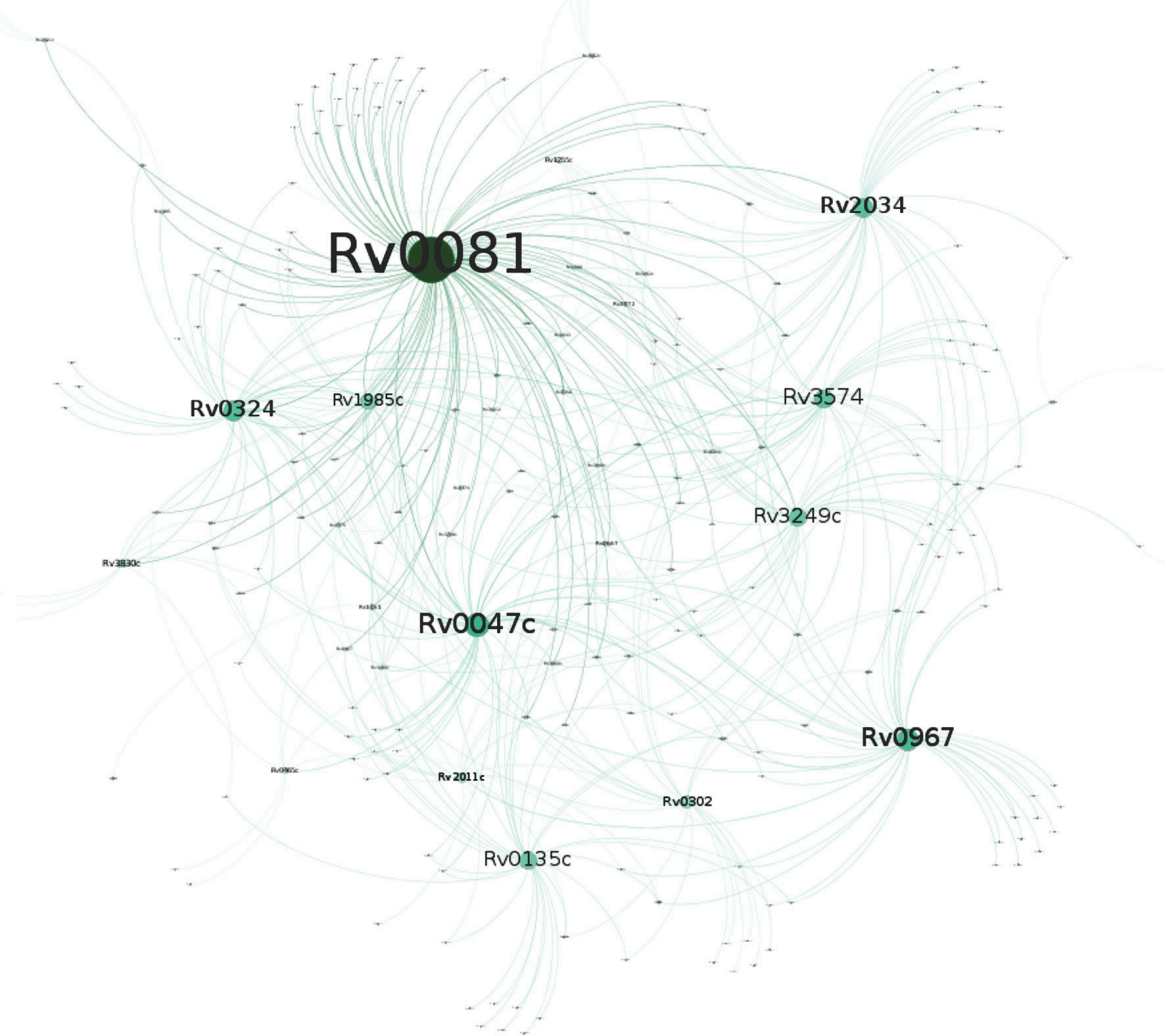
The hub of hypoxia in dextrose. The network represents genes over-expressed in DNRP1 vs. DE that interact with these TF according to the Minch's data (2015). The size of the circles is related with the number of genes controlled by the respective TF.

**Figure 4:**
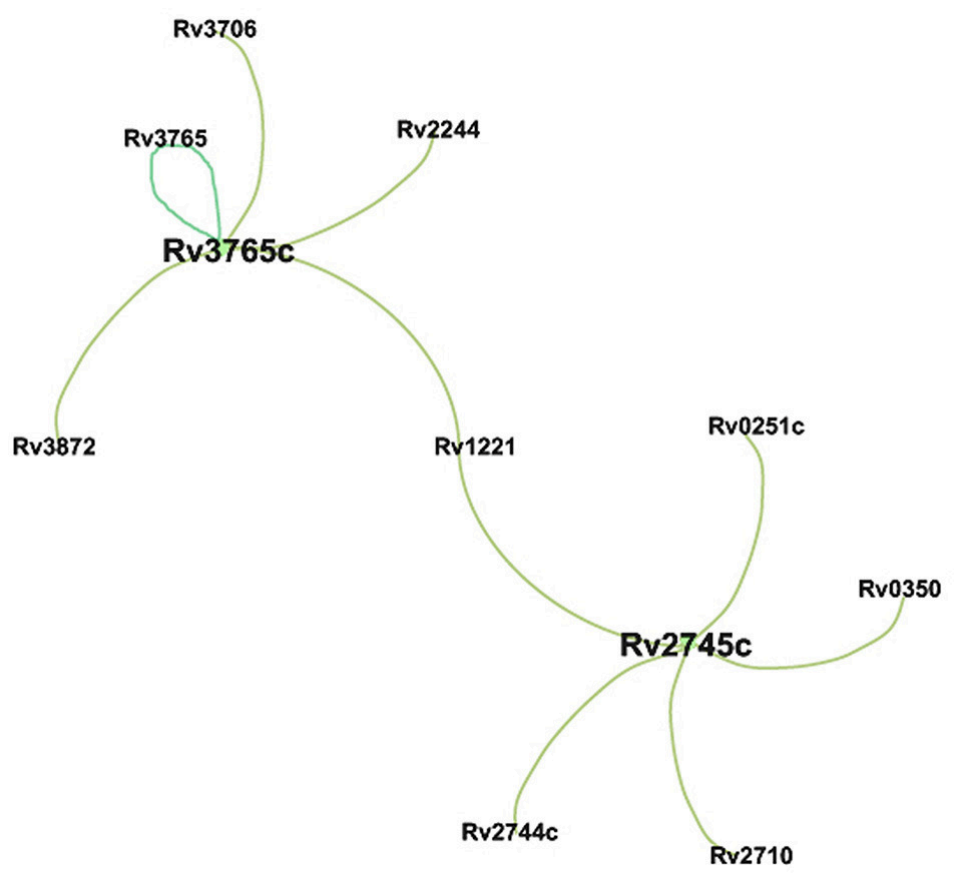
Over-expressed TF identified in FNRP1 *vs*. FE. Genes over-expressed in the FNRP1 *vs*. FE that interact with Rv3765c, according to the Minch's data (2015). Because, Minch and co-workers did not identified genes controlled by the TF Rv2745, the figure showed those described in the literature (Estorninho et al., 2010; McGillivray et al., 2014).

The genome of *Mtb* has 214 TFs (Cole et al., 1998), a relative high number considering the size of its genome. Recent work using ChIP-seq and over-expression of 206 TFs (Rustad et al., 2014; Minch et al., 2015) provided a comprehensive transcriptional map of *Mtb*. Using the data reported by these authors, we identified all the significant over-expressed TFs in the DNRP1 *v*s. DE and FNRP1 *vs*. FE (Supplementary Table 3). Then, we compared all the target genes of those TF identified using ChIP-seq by Minch's data (Minch et al., 2015) with our over-expressed genes in each of the conditions studied (Supplementary Table 4). This analysis shows that while in hypoxia in the presence of dextrose there were 37 over-expressed TFs, in the fatty acid environment only 2 TFs were over-expressed (Figures 3, 4 and Supplementary Table 4). The *Rv0081* gene, a central regulatory gene in response to hypoxia in the presence of dextrose (Galagan et al., 2013), was also the regulatory master in our DNRP1 model, confirming its pivotal role in controlling *Mtb* adaptation to low oxygen in dextrose. However, this regulator was not over-expressed in hypoxia when LC-FA was present (See Supplementary Table 3). This result could indicate that the adaptation machinery of the bacilli is already prepared to support a hypoxic stress when *Mtb* uses lipids as carbon source. The high number of TFs that were upregulated in the DNRP1 *vs*. DE condition (Figure 3 and Supplementary Table 4) indicates the strong adaptive changes required for the tubercle bacilli upon entrance to hypoxia when dextrose is the carbon source.

Only two TFs were upregulated comparing FNRP1 *vs*. FE: *Rv3765c* (*tcrX*), a two component transcriptional regulator (Bhattacharya and Das, 2011) and *Rv2745c* (*clgR*), which encodes a transcriptional protein involved in the regulation of proteases and chaperons (McGillivray et al., 2014). These data demonstrate that the adaptation to hypoxia in the presence of LC-FA supposes a minor regulatory change to the tubercle bacilli. Our data suggest that the relation of ClgR with hypoxia is maintained also in the presence of lipids. A complete set of data concerning TFs and its regulated genes are showed in Supplementary Table 4.

In our study, we detected several sigma factors over-expressed during the early hypoxic stage NRP1 in cultures including both carbon sources (DNRP1 and FNRP1) relative to exponential cultures (DE and FE) (Table 2). Up to four sigma factors were over-expressed in DNRP1 compared to DE. Two of them (*sigB* and *sigE*) were also over-expressed in FNRP1 relative to FE. It can be inferred that those two sigma factors are key in the early adaptation to hypoxia of *Mtb* independently of the carbon source. The fact that *sigE* was the sigma factor with the higher level of over-expression in both hypoxia models also supports its pivotal role in the early response to low oxygen by *Mtb* (Table 2). In addition, no significant changes were detected in the level of expression of sigma factors when comparing early (NRP1) with late (NRP2) hypoxia conditions in both carbon sources (see Supplementary Table 3) which suggests that sigma factors participate in the initial adaptation to hypoxia of *Mtb*, while remaining unchanged when bacteria face a lower level of oxygen.

**Table 2:** Expression of sigma factors in the LC-FA model of hypoxia.

**Comparison**	**Sigma factor**	**RPKM DNRP1**	**RPKM DE**	**FDR**	**RPKMs ratio**
DNRP1 > DE	*SigB*	404	105	1.67E-69	3.85
	*SigH*	133	41	4.37E-20	3.24
	*SigF*	14	5	0.025	2.80
	*SigE*	315	43	7.12E-76	7.33
FNRP1 > FE	*SigE*	47	8	5.00E-05	5.88
	*SigB*	103	43	1.47E-03	2.40

*DE, Dextrose exponential; DNRP1, Dextrose early hypoxia; FE, LC-FA exponential; FNRP1, LC-FA early hypoxia*.

### Small RNAs Involved in the Fatty Acid Model of Hypoxia

**Figure 5:**
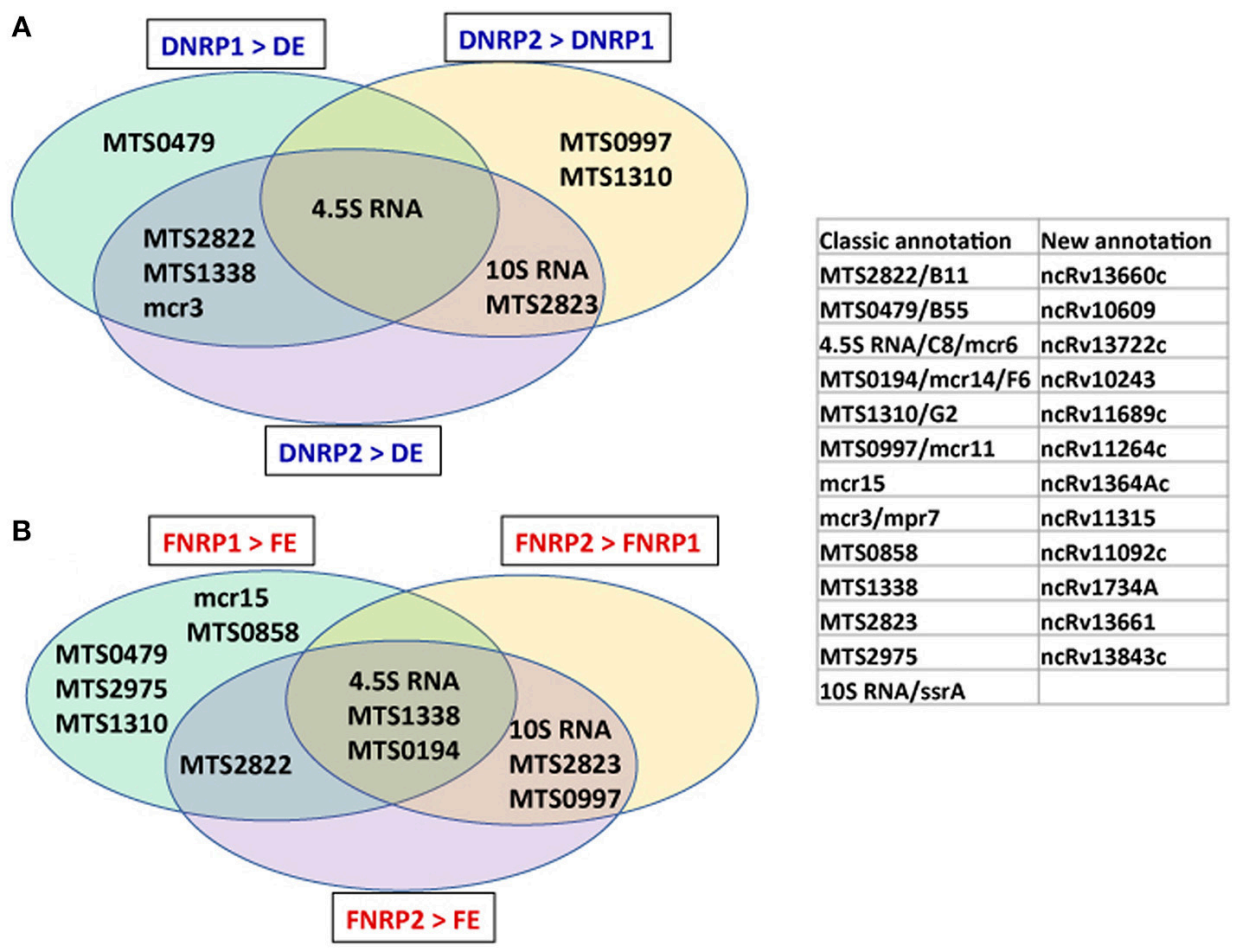
Venn diagram showing the small RNAs with significant high expression during the adaptation of *Mtb* to hypoxia in Dextrose (DNRP2>DNRP1>DE, blue letters) and in LC-FA (FNRP2>FNRP1>FE, red letters). The enclosed table contains the new annotation for the sRNAs (Lamichhane et al., 2013). The shadowed boxes indicate those sRNAs shared between carbon sources. **(A)** Dextrose (DNRP2 > DNRP1 > DE, blue letters) and in **(B)** LC-FA (FNRP2 > FNRP1 > FE, red letters).

The high level of IGRs expression detected when the tubercle bacilli enter into hypoxia (Table 1) prompted us to analyse the contribution of small RNAs (sRNAs), including non-coding RNAs (ncRNAs) in the process. The sRNAs participating in the adaptation of *Mtb* to hypoxia in the two cultured conditions studied are summarized in Figure 5. Nine and 12 sRNAs showed over-expression in the *Mtb* adaptation to hypoxia in the presence of dextrose and LC-FA, respectively (Figure 5 and Supplementary Table 5). Independently of the carbon source, the 4.5S RNA was over-expressed in all hypoxia conditions. Two more ncRNAs showed a significant higher expression only in the presence of LC-FA: MTS1338 and MTS0194 (Figure 5). Several ncRNAs seemed to have an important role in early and late hypoxia in the presence of dextrose (DNRP1 *vs*. DE and DNRP2 *vs*. DE). These are mcr3 and MTS2822 (Figure 5). Of note, MTS2822 was also over-expressed in conditions with LC-FA as carbon source (FNRP1 *vs*. FE and FNRP2 *vs*. FE) (Figure 5 and Supplementary Table 5).

Finally, significant higher expression of some sRNAs was also related to the adaptation to late hypoxia (Figure 5). MTS2823 and the stable 10S RNA were detected with significantly high expression in NRP2 in both carbon sources. In addition, MTS0997 was highly expressed in late hypoxia only when LC-FA was the carbon source. MTS2823 is a well-known ncRNA, whose levels of expression highly increased when the bacilli enter stationary phase in LC-FA (Rodríguez et al., 2014) and has also been linked to the slow-down of the *Mtb* growth (Arnvig and Young, 2012).

### Toxin–Antitoxin Systems Involved in the Fatty Acid Model of Hypoxia

Only two toxins were detected with significant higher expression during the adaptation of *Mtb* to hypoxia, with LC-FA as carbon source (FNRP1 and FNRP2; Table 3). Conversely, up to four complete TA systems, three single toxins and one single antitoxin were over-expressed when hypoxic cultures included dextrose (DNRP1 and DNRP2; Table 3). These results suggest that TA modules play a major role in the adaptation of the tubercle bacilli to hypoxia when dextrose was the carbon source but not in the presence of LC-FA. Members of the *vapBC* family were upregulated in dextrose, namely *vapB10* and *vapC37* in DNRP1, as well as the toxin *vapC20* in DNRP2 (Table 3). Of note, this system has been previously associated to hypoxia in *Mtb* (Fernandez-Garcia et al., 2016) and might have a relevant function during late hypoxia in dextrose. The ParDE2 system was suggested to be a main participant in the inhibition of bacterial growth in the related bacteria *Mycolicibacterium smegmatis* (Oren and Garrity, 2018), indicating a putative role of this TA system in dormancy and stress survival (Gupta, 2009). Our results showed that ParDE1 instead or ParDE2 could play this role in the adaptation of *Mtb* to hypoxia in dextrose (Table 3). Two of the three members of the *HigBA* family (High inhibition of growth) were also over-expressed in DNRP1 compared to DE (Table 3). The *higBA1* gene was detected with significant higher expression in DNRP1 compared to DE. HigBA1 is a member of the tripartite system TAC. This module contains three components, toxin, antitoxin, and a chaperone (HigBA1+Chaperone) (Sala et al., 2014). TAC is considered one of the main contributors to the *Mtb* survival under stress situations (Fernandez-Garcia et al., 2016), including hypoxia (Ramage et al., 2009). On the other hand, *higBA2* was also upregulated in DNRP1, suggesting for the first time that adaptation to hypoxia could be a putative function for the HigBA2 system. We only detected the involvement of a single toxin member of the *MazEF* family in DNRP1 (Table 3) in agreement with the previous data that described the connection of these TA family with persistence in the presence of antibiotics (Singh and Barry, 2010). Contrary to the adaptation to hypoxia in the presence of dextrose, only two unclassified toxins were upregulated in LC-FA during hypoxia, it can be inferred, therefore, that TA modules do not participate in the survival of *Mtb* to hypoxia in this LC-FA environment.

**Table 3:** Expression of genes coding for toxin-antitoxin systems in the LC-FA model of hypoxia.

	**TA systems**	**RPKM**	**RPKM**	**FDR**	**RPKMs ratio**
DNRP1 > DE		**DNRP1**	**DE**	
	Rv0837c toxin	11	1	0.003	11
	Maz F5 toxin	13	2	0.005	6.5
	Vap C37 toxin	13	4	0.03	3.25
	Vap B10 antitoxin	38	15	1.08E-04	2.53
	Par E1 toxin	21	9	0.0169	2.33
	Par D1 antitoxin	44	16	8.72E-06	2.75
	Hig B2 toxin	48	16	1.02E-06	3
	Hig A2 antitoxin	36	16	5.93E-04	2.25
	TAC Hig B1 toxin	29	2	2.47E-08	14.5
	TAC Hig A1 antitoxin	34	8	3.98E-06	4.25
	TAC chaperone	12	1	0.002	12
	Rv2034 toxin	21	7	0.003	3
	Rv2035 antitoxin	61	6	6.13E-16	10.17
		**DNRP2**	**DE**	
DNRP2 > DE	Vap C20 toxin	19	4	1.41E-15	4.75
		**DNRP2**	**DNRP1**	
DNRP2 > DNRP1	Vap C20 toxin	19	6	6.11E-10	3.17
		**FNRP1**	**FE**	
FNRP1 > FE	Rv3188 toxin	20	4	0.041	5
		**FNRP2**	**FNRP1**	
FNRP2 > FNRP1	Rv0837c toxin	4	0	0.037	4

*DE, dextrose exponential; DNRP1, dextrose early hypoxia; DNRP2, dextrose late hypoxia; FE, LC-FA exponential; FNRP1, LC-FA early hypoxia; FNRP2, LC-FA late hypoxia*.

### Gene Signature of Hypoxia Under the Two Different Conditions Tested: Dextrose and LC-FA

To gain insight into the metabolic route taken for the tubercle bacilli to survive to a hypoxic stress, we analyzed the genes with higher expression shared between the four hypoxic conditions tested: NRP1 *vs*. exponential and NRP2 *vs*. NRP1 of *Mtb* growing in dextrose as well as growing in LC-FA (Table 4 and Supplementary Table 6). We found 47 genes with higher differential expression under hypoxia in all conditions, those genes were all over-expressed in DNRP1 in relation to DE with the exception of the gene *Rv2554c*; only eight genes were over-expressed in DNRP2 in relation to DNRP1 (Table 4). On the other hand, 21 and 22 genes were over-expressed in conditions comparing FNRP1 with FE and FNRP2 with FNRP1, respectively. This result again suggests that *Mtb* undertake a strong metabolic change upon entrance to hypoxia when dextrose is the carbon source, and a more gradual change in their adaptation to hypoxia in the presence of LC-FA. Most of the genes with significant high expression in DNPR1 were shared with any of the other conditions tested. Five genes were only detected in hypoxia when dextrose was the carbon source (DNRP1 and DNRP2) including *tatB* (traslocase), *PE29* and *PE20* (PE-PPE family proteins), *alaS* (Alanyl-tRNA synthetase) and the hypothetical protein coded by *Rv3205* (Table 4). Interestingly, about half of the genes over-expressed in early hypoxia in dextrose (DNRP1 *vs*. DE) were also over-expressed in late hypoxia in the presence of LC-FA (FNRP2). One of those genes was *ici*A, member of the LysR family, involved in regulation of the chromosome replication. That result suggests that the level of stress found by the bacteria in dextrose at low level of hypoxia was only reached at high level of hypoxia when the bacilli grew in the lipid environment tested, in agreement with the results found in the analysis of functional categories (Figures 2A,B).

**Table 4:** Hypoxia signature genes in dextrose and LC-FA.

**Gene tag**	**Gene name**	**FDR value**				**Gene function**
		**DNRP1 > DE**	**DNRP2 > DNRP1**	**FNRP1 > FE**	**FNRP2 > FNRP1**
Rv0079		1.76E-05			3.26E-39	Dormancy associated translation inhibition
Rv0103	*ctpB*	6.11E-08		6.49E-03		Cation-transporter P-type ATPase B
Rv0122		5.72E-04			1.90E-07	Hypothetical protein
Rv0188		5.33E-14			7.53E-04	Transmembrane protein
Rv0251c	*hsp*	9.24E-232		1.19E-04		Heat shock protein hsp
Rv0275c		2.82E-04			5.52E-10	TetR family transcriptional regulator
Rv0350	*dnaK*	1.62E-68		3.10E-02		Molecular chaperone DnaK
Rv0628c		3.30E-02			1.16E-23	Hypothetical protein
Rv0678		6.76E-13			5.11E-09	Hypothetical protein
Rv0754	*PE_PGRS11*	4.64E-08			3.30E-04	PE-PGRS family protein
Rv0791c		2.00E-23			3.86E-05	Hypothetical protein
Rv0792c		2.50E-58			2.54E-09	GntR family transcriptional regulator
Rv0826		2.46E-11			1.80E-02	Hypothetical protein
Rv0837c		3.00E-03			2.10E-02	Hypothetical protein
Rv0968		8.21E-108			3.21E-54	Hypothetical protein
Rv0969	*ctpV*	3.76E-57			1.25E-54	Metal cation transporter P-type ATPase CtpV
Rv0983	*pepD*	1.14E-52		5.38E-03		Serine protease PepD
Rv0991c		4.59E-79		6.40E-04		Serine-rich protein
Rv1221	*SigE*	7.12E-76		5.00E-05		RNA polymerase sigma factor *SigE*
Rv1224	*tatB*	6.27E-17	3.37E-45			Sec-independent translocase
R1460		1.63E-05			8.00E-03	Transcriptional regulatory protein
Rv1462		2.49E-30			6.42E-07	Hypothetical protein
Rv1535		4.06E-05		4.17E-05		Hypothetical protein
Rv1801	*PPE29*	8.55E-05	7.07E-08			PPE family protein
Rv1806	*PE20*	2.03E-09	3.01E-17			PPE family protein
Rv1807	*PPE31*	1.19E-18	3.40E-25	2.66E-04		PPE family protein
Rv1831		1.76E-04	2.89E-262	1.17E-03		Hypothetical protein
Rv1985c	*iciA*	2.04E-08			2.88E-11	LysR-family
Rv2050		1.23E-67		3.71E-03		Hypothetical protein
Rv2169c		2.07E-04		1.43E-03		Transmembrane protein
Rv2516c		4.00E-20			1.90E-02	Hypothetical protein
Rv2554c			2.14E-78		1.58E-09	Holliday junction resolvase-like protein
Rv2555c	*alaS*	7.94E-04	2.53E-253			Alanyl-tRNA synthetase
Rv2623	*Tb31.7*	7.38E-158			6.77E-67	Hypothetical protein
Rv2694c		7.50E-88		9.61E-06		Hypothetical protein
Rv2699c		1.20E-146		2.58E-05		Hypothetical protein
Rv2710	*sigB*	1.67E-69		1.47E-03		RNA polymerase sigma factor SigB
Rv2744c	*35kd_ag*	1.56E-110		5.42E-03		Hypothetical protein
Rv2745c	*clgR*	2.48E-297		9.51E-03		Transcriptional regulatory protein
Rv3205c		1.03E-05	8.37E-54			Hypothetical protein
Rv3270	*ctpC*	2.67E-22			2.16E-04	Metal cation-transporting P-type ATPase C CtpC
Rv3289c		2.30E-90			1.07E-22	Transmembrane protein
Rv3290c	*lat*	5.21E-76			1.59E-34	L-lysine aminotransferase
Rv3417c	*groEL*	1.13E-15		2.66E-05		Molecular chaperone GroEL
Rv3418c	*groES*	4.60E-13		2.36E-57		Co-chaperonin GroES
Rv3461c	*rpmJ*	5.00E-03		8.32E-04		50S ribosomal protein L36
Rv3679		1.13E-11		2.38E-10		Anion transporter ATPase
Rv3872	*PE35*	2.50E-05		4.88E-07		PE family-like protein

*DE, dextrose exponential; DNRP1, dextrose early hypoxia; DNRP2, dextrose late hypoxia; FE, LC-FA exponential; FNRP1, LC-FA early hypoxia; FNRP2, LC-FA late hypoxia*.

A complete set of RPKM data concerning genes included in Table 4 are showed in Supplementary Tables 3, 6.

#### Quantitative RT-PCR of Selected Genes

The over-expression of 13 selected genes was further confirmed by quantitative reverse transcription PCR (qRT-PCR) (Supplementary Figure 1). Representative over-expressed genes were selected from those coding for sigma factors (SigB and SigE) transcriptional regulatory factors (Rv0081, ClgR and TcrX) small RNAs (10SRNA, MTS0194, and MTS2823) and toxin-antitoxin systems (VapC20, HigB1, and HigA1). The results of the qRT-PCR are in agreement with those observed in the transcriptomic assays.

## Discussion

*Mtb* must face different environmental changes and stresses to survive and establish a long-lasting latent infection (Getahun et al., 2015; Ehrt et al., 2018). Low level of oxygen is considered one of the main characteristics involved in *Mtb* adaptation to the host environments (Wayne and Hayes, 1996) either during active disease or during latent infection (Flentie et al., 2016). Although it is now well-accepted that hypoxia is a rather simplistic way to describe those environments, the low level of oxygen is nowadays one of the more studied stresses to which the tubercle bacilli have to face for survival inside the host. Taking into consideration that *Mtb* is surrounded by LC-FA inside the host (Lehninger et al., 2008; Santucci et al., 2016) and that hypoxia is also an environmental component under those conditions, we interrogate the transcriptomic pathways of the bacilli to adapt and survive from a standard level of oxygen to early hypoxia (1% oxygen) and late hypoxia (0.06% oxygen) in the presence of LC-FA. On the basis of the culture media tested by Rodriguez and co-workers (Rodríguez et al., 2014) we implemented the Wayne's model of hypoxia and collected cells for global transcriptomic studies at early (NRP1) and late (NRP2) hypoxia levels.

### Global Adaptation to Hypoxia in a LC-FA Culture Media

From a global perspective we found similarities and differences in the metabolic pathways undertaken by the bacilli when comparing hypoxia in the presence of dextrose vs. conditions including LC-FA as main carbon source. In agreement with our previous results (Rodríguez et al., 2014), we observed an increased level of reads mapping to IGRs in LC-FA (Table 1), suggesting a relevant role for small RNAs and other IGRs when LC-FA are the culture media. We also observed striking differences when analyzing the functional categories of the genes of interest during the adaptation to hypoxia comparing both carbon sources. The abundance of genes belonging to the functional category of information pathways showed opposite trends in both conditions, being reduced in DNRP1 *vs*. DE but increased in FNRP1 *vs*. FE (Figure 2). This could indicate that the basic metabolic activity, important for the bacilli under hypoxia in LC-FA, was not that relevant when dextrose was the carbon source. A similar result was previously described when cholesterol was added to LC-FA media (Aguilar-Ayala et al., 2017), which therefore could represent a general condition associated to the *Mtb* adaptation to early hypoxia in lipid environments. According to these authors, the addition of cholesterol makes the bacteria more metabolically active in NRP1. In fact, the addition of cholesterol increased the percentage of reads mapped to CDS in NRP1 comparing to those found in the absence of cholesterol (Aguilar-Ayala et al., 2017) (Table 1). In relation to this, it has been found that a high-cholesterol diet was related to higher risk of active TB (Soh et al., 2016).

### Regulatory Factors Involved in the Adaptation to Hypoxia in LC-FA

We observed that the number of over-expressed genes encoding regulatory proteins was different between hypoxic cultures supplemented with both carbon sources (Figure 2, Supplementary Table 3). The participation of those genes was relevant in early hypoxia in dextrose (Figure 3) and less relevant in LC-FA (Figure 4). Among sigma factors, *sigE* is one of the most studied of *Mtb* and it has been proposed as a central regulator of the stress response of the pathogen (Datta et al., 2011). Recently it was demonstrated that SigE has a major role in determining the level of basal tolerance of *Mtb* to antitubercular drugs (Pisu et al., 2017). We detected that *sigE* and *sigB* increased upon entrance to hypoxia regardless the nature of the carbon source, reinforcing the role described for *sigE* as a central regulator of hypoxia in *Mtb* (Du et al., 2016) a condition associated to drug tolerance. Our results indicate that *sigB* could be an additional component of this central system (Table 2). Supporting this notion is the fact that both *sigE* and *sigB* were found over-expressed during persistence (Du et al., 2016; Flentie et al., 2016) another stress condition for the bacilli.

We found that the central hypoxia regulator in dextrose, namely *Rv0081* (Galagan et al., 2013) although over-expressed in NRP1 in both carbon sources, play a main role in DNRP1 (Figure 3) compared to FNRP1 (Figure 4) where other two transcriptional factors, namely TcrX and ClgR, seemed to have a main participation (Supplementary Figure 1 and Table 3).

Aiming to define a hypoxic signature (Table 4), we identified five transcriptional regulators with significantly high expression in DNRP1. Four of those have also significant high expression in FNRP2 and the remaining one, namely *clgR* (*Rv2745*), is also highly expressed in FNRP1. According to our data, this regulator may play a main role in the early adaptation to hypoxia in the LC-FA environment (Figure 4 and Supplementary Figure 1). ClgR is a transcriptional regulatory protein that has been involved in the maintenance of the membrane integrity of *Mtb* during response to stress (Veatch and Kaushal, 2018). In fact, *clgR* is induced in *Mtb* upon several stressful conditions including redox stress by diamide, SDS, hypoxia and low pH (McGillivray et al., 2014). In addition, a main role in the reactivation of bacilli after dormancy-induced by hypoxia was also assigned to this regulator (McGillivray et al., 2015). Our data in DNRP1 (Supplementary Table 3) were in accordance with the description of ClgR as negative regulator of Clp proteases in dextrose (McGillivray et al., 2015). This result could be related with the increased level of toxin-antitoxin activity found in dextrose (Table 3) through inactivation of antitoxins by Clps. On the contrary, the expression of Clp proteases did not change upon entrance to FNRP1 (Supplementary Table 3) suggesting a different role for ClgR in LC-FA. We found that the tubercle bacillus is in a reductive stress under LC-FA lipid environment (Rodríguez et al., 2014); therefore, the increased expression found for this regulator in FNRP1 (Figure 4 and Supplementary Table 3) could indicate that its role in lipid media was related with their participation in redox stresses. In agreement with this is the previous finding that the induction of *clgR* during redox stress did not result in the induction of *clp* genes (McGillivray et al., 2014). Our results highlight, for the first time, the influence of the environment in the complex regulatory activity of ClgR (Veatch and Kaushal, 2018).

One of the common regulatory factors between DNRP1 and FNRP2 was *iciA* (*Rv1985c*) (Figure 3 and Table 4). IciA is an *in vitro* replication initiation inhibitory protein (Kumar et al., 2009; Zhou et al., 2010; Marcus et al., 2016) probably involved in the entrance into periods of quiescence through the control of the chromosomal replication. Induction of *iciA* has been previously described during nutrient starvation (Zhou et al., 2010). Interestingly, *iciA* appears to be required at early hypoxia in dextrose, but it is not required until late hypoxia in the presence of LC-FA, what suggests, again, that that lipid environment is a less stressful condition for the tubercle bacilli. Notably, a single gene (*Rv2554c*) was only over-expressed in NRP2 regardless the carbon source, besides showing significant high expression in DNRP2 compared to FNRP2. This gene encodes a holliday junction resolvase-like protein, suggesting the implication of the reparation of double-strand breaks process in late hypoxia.

Other interesting regulators involved in the adaptation to hypoxia in LC-FA are sRNAs. These regulatory molecules were more relevant in the adaptation when LC-FA was the carbon source (Figure 5 and Supplementary Table 5). It is worth to mention that several ncRNAs with high expression in the adaptive pathway to hypoxia had been previously associated to slow down growth by *Mtb* (Arnvig and Young, 2012). This was the case of MTS1338, MTS0997, MTS0194, and MTS2823 (Figure 5) showing this activity as an apparently common feature of ncRNAs in this bacillus. The over-expression of either MTS1338 or MTS0194 also causes slow-down of the *Mtb* growth rate when the bacteria is cultured in the low-potassium *in vitro* dormancy model (Haning et al., 2014; Ignatov et al., 2014). On the other hand, the over-expression of MTS2823 causes down regulation of many genes involved in the energy metabolism (Arnvig and Young, 2012).

The MTS0194 is induced upon hydrogen peroxide and acid stress, two conditions that *Mtb* faces inside macrophages during active infection (Arnvig and Young, 2012). Interestingly, the cooper-inducible regulatory protein CsoR binds MTS0194 (Minch et al., 2015). CsoR loses its repressor activity under high levels of Cu (Marcus et al., 2016). The high level of expression found for MTS0194 in FNRP1 and FNRP2 suggests the loss of that repressor activity under hypoxia in LC-FA (see Supplementary Table 3) with increasing level of expression of MTS0194 as a consequence (see Supplementary Table 5 and Figure 1). This result could indicate that hypoxia in LC-FA represents a condition closer to that described inside the mycobacterial phagosome, where a high level of Cu is present (Marcus et al., 2016).

Regardless the culture media, some stable RNAs were detected with high expression in early and late hypoxia. The 4.5S RNA showed very high level of expression both in early and late hypoxia, being higher when *Mtb* is in dextrose compared to LC-FA (Supplementary Table 5). On the other hand, the 10S RNA also showed high level of expression in late hypoxia. These two RNAs are involved in key metabolic activities that help to the adequate function of the protein synthesis by the cell. The 4.5S RNA has been identified in *Mtb* as part of the signal recognition particle (SRP) together to the Ffh protein, and it is involved in the recognition of signal peptides emerging from the ribosomes, assisting the transport from the cell toward their functional compartments (Arnvig and Young, 2009; Palaniyandi et al., 2012). The 10S RNA, encoded by the gene *ssrA*, has been identified as stable tmRNA participating in the trans-translation in *Mtb* (Personne and Parish, 2014). The tmRNAs are responsible for recycling stalled ribosomes and thus ensuring their availability for protein synthesis, avoiding the accumulation of abnormal proteins by the bacteria. Different from other bacteria that have several systems to recover stalled ribosomes, *Mtb* has only the system represented by tmRNA and the accessory protein SmpB (Rv3100c). Contrary to *smpB*, the *ssrA* gene is essential in *Mtb* (Personne and Parish, 2014). Our data suggest for the first time the key role that both small RNAs may play in the survival of the tubercle bacillus during hypoxia. Our data also confirm the main contribution of small stable RNAs in the adaptation of *Mtb* to hypoxia, being that role particularly relevant in the dormancy-like lipid environment represented by LC-FA.

### Toxin-Antitoxin Systems and the Adaptation to Hypoxia

TA systems are considered relevant for persisting bacteria, helping their survival under stress conditions through the selected downregulation of targeted genes (Gerdes and Maisonneuve, 2012). In addition, the expression of these TA systems is regulated by environmental cues, which are relevant in the context of persistent infections, such as those caused by *Salmonella enterica, Helicobacter pylori*, and *Mtb* (De la Cruz et al., 2013; Cárdenas-Mondragón et al., 2016; Slayden et al., 2018). Our results suggest that TA modules play a role in the adaptation of *Mtb* to hypoxia in dextrose (Table 3) similarly to the survival mechanism switched on by the bacteria in a persistence-related stress condition (Butt and Titball, 2016). On the contrary, TA modules do not have a relevant participation in hypoxia in LC-FA (Table 3). It can be inferred that hypoxia does not represent a relevant stress for the tubercle bacilli when *Mtb* uses LC-FA as a carbon source. Interestingly, Aguilar-Ayala and co-workers (Aguilar-Ayala et al., 2017) detected participation of complete TA systems in NRP1 condition when cholesterol was added to the LC-FA medium. This result again suggested that cholesterol, a metabolic source of propionate, makes this last condition more toxic to the bacilli (Galagan, 2014) and could represent a stress environment that requires a decreased cell activity mediated by TA modules for survival.

## Concluding Remarks

*Mtb* is considered to be well-adapted to their human host, being latency a hallmark of this adaptation. During latency, the tubercle bacilli reside inside foamy macrophages filled of lipid bodies, where triacylglycerols and hypoxia are main environmental conditions. Our results disclosed the regulatory pathways undergone by *Mtb* to adapt to such conditions and represent and important source of information to understand the path to reach the quiescent phenotype established during latency.

The transcriptional adaptation of *Mtb* to hypoxia, in the *in vitro* model of dormancy represented by even LC-FA, detected the implication of different key stakeholders in the process, such as the scarce participation of TA modules or the role played by small RNAs. Interestingly, the addition of cholesterol reverts that transcriptional machinery of dormancy to one closer to that in dextrose, this indicating that cholesterol *in vitro* could be a condition more related to active growth than to dormancy.

When LC-FAs were present in the culture medium, instead of dextrose, *Mtb* was gradually adapted to hypoxia, showing a less-stressful transcriptome, closely related to the dormancy-adapted condition. The low level of stress-response showed by *Mtb* during their adaptation to hypoxia in LC-FA, together to the participation of genes known to be associated to the environment inside the granuloma, demonstrates that the bacterium appears to be already adapted and strongly suggests the relationships of those lipid conditions with the environment found during latent infection.

## Author Contributions

The study was conceived and designed by MG, PD, JG-y-M, JA, and JR. Experiments were conducted by AH-R and JR. Bioinformatic work flow was conducted by JA and MM. Data interpretation was performed by MG, PD, JG-y-M, LG-M, JR, MA, MM, and RP-R. Manuscript was written by PD, MG, JG-y-M, MM, and revised by LG-M, JR, MA, and RP-R. All authors approved the manuscript.

### Conflict of Interest Statement

The authors declare that the research was conducted in the absence of any commercial or financial relationships that could be construed as a potential conflict of interest.

## Supplementary Material

Supplementary Figure S1 Validation by qRT-PCR of selected genes over-expressed under hypoxia. **(A)** Number of copies corresponding to the several conditions and carbon sources tested. Data were normalized to the number of copies of the *rrs* gene (16S rRNA). Data are expressed as log_10_ of the number of copies per microgram of RNA. **(B)** Fold change of the number of copies according to the adaptation to hypoxia in each of the carbon sources applied. aExponential phases, DE and FE; Early hypoxia, DNRP1 and FNRP1; Late hypoxia, DNRP2 and FNRP2. Data corresponding to each of the two carbon sources used are indicated by range of color: blue, dextrose; ochre, LC-FA.

Supplementary Table S1 Primers used for qRT-PCR.

Supplementary Table S2 Common genes overexpressed comparing TWO THREE and FOUR conditions.

Supplementary Table S3 RPKM values and functional categories of overexpressed *M. tuberculosis* genes.

Supplementary Table S4 Genes regulated by transcriptional factors overexpressed in DNRP1 vs. DE (see text for explanation).

Supplementary Table S5 Level of expression of non-coding RNAs in the LC-FA model of hypoxia.

Supplementary Table S6 RPKMs ratio of genes belonging to the hypoxia signature.

## Abbreviations

DE: Dextrose exponential growth phase
DNRP1: Dextrose non-replicative persistence 1
DNRP2: Dextrose non-replicative persistence 2
LC-FA: long chain fatty acids
FE: long chain fatty acid exponential growth phase
FNRP1: long chain fatty acid non-replicative persistence 1
FNRP2: long chain fatty acid non-replicative persistence 2.
