# Supplementary figures and images for: Hypoxia Is Not a Main Stress When Mycobacterium tuberculosis Is in a Dormancy-Like Long-Chain Fatty Acid Environment
Source: Front Cell Infect Microbiol. 2019 Jan 9;8:449. doi: 10.3389/fcimb.2018.00449 (PMC6333855; doi:10.3389/fcimb.2018.00449)

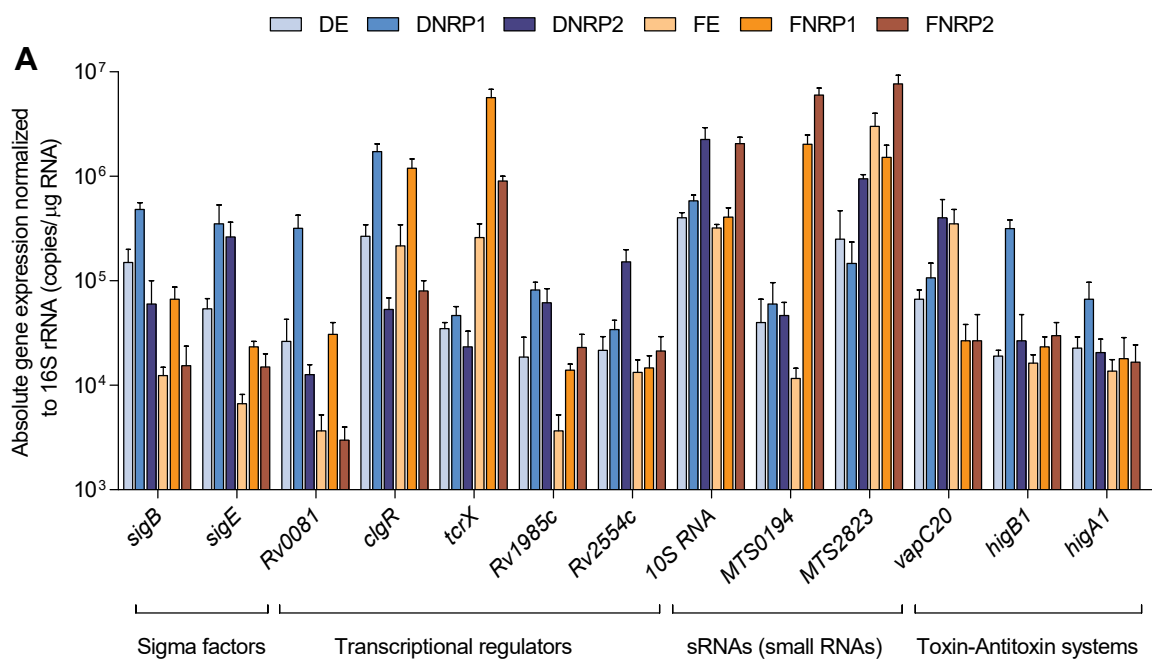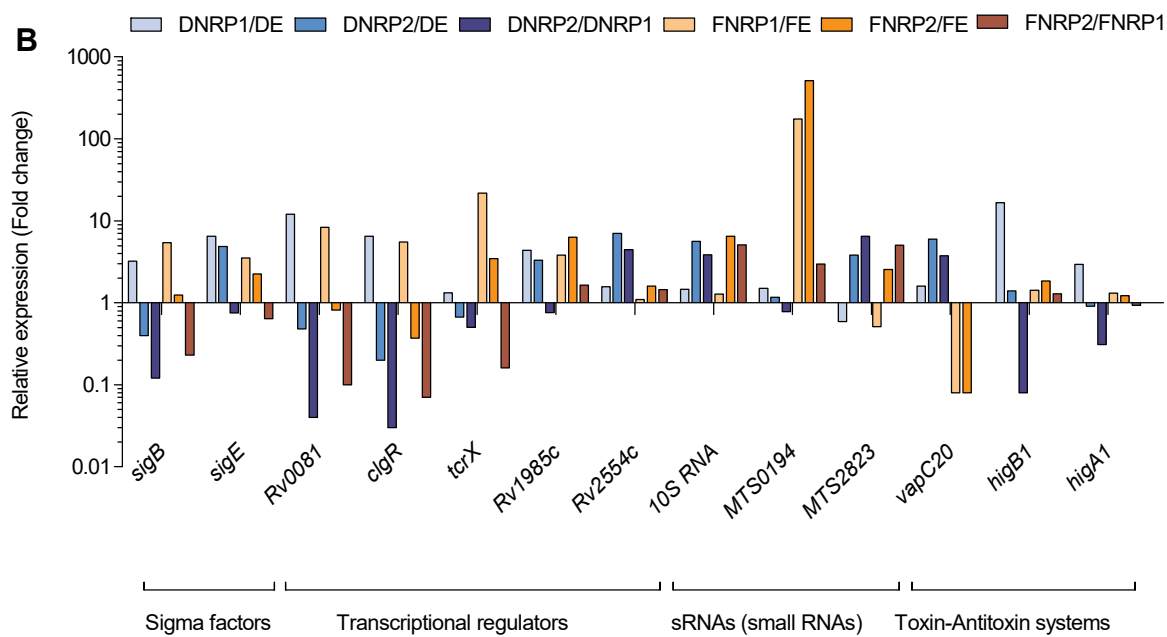

Supplement: Supplementary Figure S1 — Validation by qRT-PCR of selected genes over-expressed under hypoxia. (A) Number of copies corresponding to the several conditions and carbon sources tested. Data were normalized to the number of copies of the rrs gene (16S rRNA). Data are expressed as log10 of the number of copies per microgram of RNA. (B) Fold change of the number of copies according to the adaptation to hypoxia in each of the carbon sources applied. aExponential phases, DE and FE; Early hypoxia, DNRP1 and FNRP1; Late hypoxia, DNRP2 and FNRP2. Data corresponding to each of the two carbon sources used are indicated by range of color: blue, dextrose; ochre, LC-FA. [file Image_1.pdf]
