# Supplementary material for: Hypoxia Is Not a Main Stress When Mycobacterium tuberculosis Is in a Dormancy-Like Long-Chain Fatty Acid Environment
Source: Front Cell Infect Microbiol. 2019 Jan 9;8:449. doi: 10.3389/fcimb.2018.00449 (PMC6333855; doi:10.3389/fcimb.2018.00449)
Supplement: Supplementary Table S1 — Primers used for qRT-PCR. [file Table_1.DOCX]

| **Target** | **Primers sequence (5´ to 3´)** | **Product name** | **Tm (ºC)** | **Size of the product (pb)** | **Reference** |
| --- | --- | --- | --- | --- | --- |
| *sigB* | F: ATCGCCGAACTGTTACACAC  R: GCCCGAATAGTTTGCCGATTTG | RNA polymerase sigma factor SigB | 59 | 139 | This study |
| *sigE* | F: TGGGAATACGGAATCGCAAC  R: ATCCTCCGAATTTGCACTGC | RNA polymerase sigma factor SigE | 58 | 76 | This study |
| \| Rv0081 \| \| --- \| | F: AGTCCGAACCGCTGTACAAG  R: CAGCAGCTCCAAAATCCTGATC | HTH-type transcriptional regulator | 60 | 83 | This study |
| \| *clgR* \| \| --- \| | F: GTGCGATTTGTACGGCTCTG  R: CAATGACGACCTTGGTGCTG | Transcriptional regulator ClgR | 59 | 150 | This study |
| \| *tcrX* \| \| --- \| | F: TTGACGTGCTGCACAAGTTG  R: ATGCTAAACGGCTTGGTGAC | Two component system regulator TcrX | 59 | 136 | This study |
| \| Rv1985c \| \| --- \| | F: ATTCGTCCAGCGCCATCTATC  R: TTACGCACCAACATGTCCTG | HTH-type transcriptional regulator | 58 | 108 | This study |
| Rv2554c | F: AGTGCTCAACGATCTTTGCG  R: ATCGGAGCCTTCTTGAGTCC | Holliday junction resolvase-like | 59 | 144 | This study |
| *ssrA* | F: AACCGCAAACTGCTGTTGC  R: AAGCTCAACCTTGACCTTGC | 10SRNA (tmRNA) | 59 | 123 | This study |
| MTS0194 | F: GATAGCCCCGTGTTGTTGTC  R: GGGTCCCCTCCCACCAG | Non coding RNA | 59 | 89 | This study |
| MTS2823 | F: TCGATCCAGAAGAGAAGGTTCG  R: TGTTCGCAATTACGCAGACC | Non coding RNA | 59 | 120 | This study |
| *vapC20* | F: CATGGGAATGGCTTGTACGC  R: TGCCTTTCTTTCGCATCACC | Toxin VapC20 | 59 | 84 | This study |
| *higB1* | F: ACATTCTGGAGTTGCGATGG  R: TTCGTCTTGGGAGTCTTCTGC | Toxin HigB1 | 58 | 124 | This study |
| *higA1* | F: TCTGTTCTGTGTGGGACTGATG  R: GCAGGAACGTGTTGAAGTAGTG | Antitoxin HigA1 | 60 | 110 | This study |
| *rrs* | F: GTAATCGCAGATCAGCAACG  R: TTCGGGTGTTACCGACTTTC | 16S Ribosomal RNA | 58 | 89 | Ares *et al*., 2017 |

**Supplementary Table S1. Primers used for qRT-PCR**
